# F.A.C.E.: Friendly And Considerate Editors

**DOI:** 10.1098/rsob.250008

**Published:** 2025-01-29

**Authors:** Jonathon Pines

**Affiliations:** ^1^ Division of Cell and Molecular Biology, Institute of Cancer Research, London, UK

**Figure:**
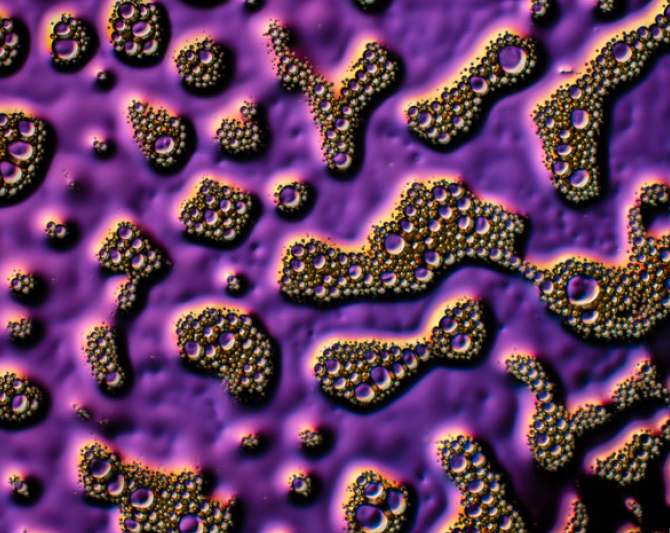

As we enter 2025, the importance of improving the level of understanding in the world is ever more apparent: understanding in the sense of increasing and communicating the sum of human knowledge, the main purpose of scientific journals; and understanding in the sense of acknowledging differences in views. For *Open Biology*, this means fair and balanced editorial decisions based on the opinions of both peer reviewers and the authors.

Our editors are clearly managing this balancing act—compared with the last quarter, submissions to *Open Biology* were up and times to decision were down, and we are proud to say that we have many returning authors—but we wish to go further to enrich our authors’ experience. This year we will be enhancing the communication between editors and authors on how to revise papers with the aim to formulate a mutually agreed structured revision plan. In this way, we will augment the journal’s reputation for clear and constructive peer review.

Constructive peer review is the core mission of *Open Biology*, and we have introduced a new feature to showcase this. We offer our reviewers the opportunity to write a ‘Spotlight’ commentary on research that they have evaluated that is particularly noteworthy for both the rigour of the science and its significance. Our editors can also select noteworthy contributions for highlighting in our Cassyni Research Seminars series that has its own dedicated channel.

We continue to build the journal as a place for discussion and debate. Our Open Questions articles highlight advances in an area of cellular and molecular biology that is developing quickly and ripe for discovery—many thanks to our Associate Editors Martha Cyert and Tin Tin Su for advocating for this initiative—and the winner of the first Open Questions competition will be announced shortly. We received 26 submissions to the competition and were so impressed by the quality of the articles that we intend to run the competition every year.

I will close by thanking all our editors, reviewers, authors and especially Buchi and the production staff, for helping to make *Open Biology* a place of harmony in 2025!

